# Continuous Particle Separation in Microfluidics: *Deterministic Lateral Displacement* Assisted by Electric Fields

**DOI:** 10.3390/mi12010066

**Published:** 2021-01-09

**Authors:** Pablo García-Sánchez, Antonio Ramos

**Affiliations:** Departamento de Electrónica y Electromagnetismo, Facultad de Física, Universidad de Sevilla, Avenida Reina Mercedes s/n, 41012 Sevilla, Spain; pablogarcia@us.es

Advances in the miniaturization of microelectromechanical systems (MEMS) [1] are revolutionizing the possibilities of sample analysis. For example, microfluidic devices enable the manipulation of tiny amounts of liquids, minimizing the consumption of samples and reagents and paving the way to portable Lab-on-a-Chip (LOC) devices for point-of-care diagnosis [2]. Pretreatments such as separation and concentration of target analytes are an essential step before assays on biological or environmental samples [3], which usually consist of a mixture of components including particles of microscopic dimensions. Therefore, the development of efficient and reliable particle separation techniques is a major challenge in the progress of LOC technologies.

An innovative strategy for continuous microparticle sorting in microfluidics is the use of deterministic lateral displacement (DLD) devices [4,5,6]. Several recent publications demonstrate its potential for separation of bioparticles [7,8,9]. DLD devices consist in microchannels containing an array of pillars with diameters around tens of microns or smaller. The pillars are arranged in rows that are slightly tilted with respect to the lateral channel walls. When a liquid with suspended particles flows through the array, it turns out that particles bigger than a critical size ($D_{c}$) bump on the pillars and deviate. Repetition of this bumping results in a net lateral displacement of the particles reaching the end of the channel. This is in contrast to the behavior of particles smaller than $D_{c}$, which zigzag around the pillars and keep moving in the direction of the fluid flow [10]. DLD devices perform binary separation mainly by size (separation based on particle deformability has also been demonstrated [11,12]). $D_{c}$ is determined by geometrical factors such as pillar radius, gap between neighboring pillars, and tilting angle of the rows, which cannot be tuned once the microchannel is fabricated.

A current topic of research is the effect of electric fields on the motion of particles in aqueous suspensions within DLD devices. The first work of electric-field assisted DLD separation was by Beech et al. [13], who inserted electrodes at the inlet and outlet of the microchannel and demonstrated that particles smaller than $D_{c}$ can be displaced upon application of ac electric fields with a frequency of 100 Hz. Thus, the particle deviation can be externally controlled via an electrical signal. More recent work is based on the application of an electric field perpendicular to the flow direction [14,15]. This is accomplished by integrating electrodes on the sides of the microchannel, reducing the electrode gap and, consequently, the amplitude of the applied voltages. Among the benefits of using lower voltages, we point out the possibility of increasing the frequency range of the ac signals up to hundreds of kHz—these frequencies are not achievable by standard voltage amplifiers if required to generate thousands of volts. Using this electrode configuration, Calero et al. [16] recently demonstrated that particle deviation at high frequencies (f>1 kHz) is caused by dielectrophoresis (DEP), i.e., the movement of particles in a nonuniform electric field caused by polarization effects [17,18]. However, for low frequencies of the applied voltages, the particles undergo electrophoresis and oscillate perpendicular to the flow direction. In this case, the particles are also deflected, and, significantly, the threshold electric field magnitude for particle deviation is much lower than for high frequencies (DEP deviation).

The physical mechanism behind particle separation at low frequencies has not been clarified yet. The smaller values of the applied electric fields suggest that another phenomenon different from DEP is responsible for this. Recent experimental work has shown the appearance of stationary flow vortices around the pillars for ac signals around hundreds of Hz and below [16,19,20]. Future work will be focused on the effect of these flows on particle separation, not only on DLD channels but also in related problems such as insulating-DEP (iDEP) devices where constrictions create non-homogeneous electric fields leading to dielectrophoretic trapping of particles [21,22].

## References

[1] Madou M.J. (2018). Fundamentals of Microfabrication: The Science of Miniaturization.

[2] Reyes D.R., Iossifidis D., Auroux P.A., Manz A. (2002). Micro total analysis systems. 1. Introduction, theory, and technology. Anal. Chem..

[3] Mariella R. (2008). Sample preparation: The weak link in microfluidics-based biodetection. Biomed. Microdevices.

[4] Huang L.R., Cox E.C., Austin R.H., Sturm J.C. (2004). Continuous particle separation through deterministic lateral displacement. Science.

[5] McGrath J., Jimenez M., Bridle H. (2014). Deterministic lateral displacement for particle separation: A review. Lab Chip.

[6] Salafi T., Zhang Y., Zhang Y. (2019). A review on deterministic lateral displacement for particle separation and detection. Nano-Micro Lett..

[7] Davis J.A., Inglis D.W., Morton K.J., Lawrence D.A., Huang L.R., Chou S.Y., Sturm J.C., Austin R.H. (2006). Deterministic hydrodynamics: Taking blood apart. Proc. Natl. Acad. Sci. USA.

[8] Holm S.H., Zhang Z., Beech J.P., Gompper G., Fedosov D.A., Tegenfeldt J.O. (2019). Microfluidic Particle Sorting in Concentrated Erythrocyte Suspensions. Phys. Rev. Appl..

[9] Liu Z., Huang F., Du J., Shu W., Feng H., Xu X., Chen Y. (2013). Rapid isolation of cancer cells using microfluidic deterministic lateral displacement structure. Biomicrofluidics.

[10] Inglis D.W., Davis J.A., Austin R.H., Sturm J.C. (2006). Critical particle size for fractionation by deterministic lateral displacement. Lab Chip.

[11] Holmes D., Whyte G., Bailey J., Vergara-Irigaray N., Ekpenyong A., Guck J., Duke T. (2014). Separation of blood cells with differing deformability using deterministic lateral displacement. Interface Focus.

[12] Xavier M., Holm S.H., Beech J.P., Spencer D., Tegenfeldt J.O., Oreffo R.O., Morgan H. (2019). Label-free enrichment of primary human skeletal progenitor cells using deterministic lateral displacement. Lab Chip.

[13] Beech J.P., Jönsson P., Tegenfeldt J.O. (2009). Tipping the balance of deterministic lateral displacement devices using dielectrophoresis. Lab Chip.

[14] Calero V., Garcia-Sanchez P., Honrado C., Ramos A., Morgan H. (2019). AC electrokinetic biased deterministic lateral displacement for tunable particle separation. Lab Chip.

[15] Calero V., Garcia-Sanchez P., Ramos A., Morgan H. (2019). Combining DC and AC electric fields with deterministic lateral displacement for micro-and nano-particle separation. Biomicrofluidics.

[16] Calero V., Garcia-Sanchez P., Ramos A., Morgan H. (2020). Electrokinetic biased Deterministic Lateral Displacement: Scaling Analysis and Simulations. J. Chromatogr. A.

[17] Pohl H.A. (1978). Dielectrophoresis: The Behavior of Neutral Matter in Nonuniform Electric Fields (Cambridge Monographs on Physics).

[18] Jones P.V., Salmon G.L., Ros A. (2017). Continuous separation of DNA molecules by size using insulator-based dielectrophoresis. Anal. Chem..

[19] Ho B.D., Beech J.P., Tegenfeldt J.O. (2020). Charge-Based Separation of Micro-and Nanoparticles. Micromachines.

[20] Calero V., Fernández-Mateo R., Morgan H., García-Sánchez P., Ramos A. (2021). Origin of Stationary Electroosmotic Flow Driven by AC Fields Around Insulators. Phys. Rev. Appl..

[21] Cummings E.B., Singh A.K. (2003). Dielectrophoresis in microchips containing arrays of insulating posts: Theoretical and experimental results. Anal. Chem..

[22] Lapizco-Encinas B.H., Simmons B.A., Cummings E.B., Fintschenko Y. (2004). Insulator-based dielectrophoresis for the selective concentration and separation of live bacteria in water. Electrophoresis.

